# HIV Infection Among Heterosexuals at Increased Risk — United States, 2010

**Published:** 2013-03-15

**Authors:** Isa J. Miles, Binh C. Le, Cyprian Wejnert, Alexandra Oster, Elizabeth DiNenno, Gabriela Paz-Bailey

**Affiliations:** Div of HIV/AIDS Prevention, National Center for HIV, STD, and TB Prevention, CDC

In 2009, an estimated 27% of human immunodeficiency virus (HIV) infections in the United States were attributed to heterosexual contact (1). During 2006–2007, as part of the data collection for the National HIV Behavioral Surveillance System (NHBS), CDC surveyed heterosexuals who lived in urban areas with a high prevalence of acquired immunodeficiency syndrome (AIDS) and found an overall HIV prevalence of 2.0% and a prevalence of 2.3% among persons with annual household incomes at or below the poverty level and 2.8% among persons with less than a high school education (2). This report summarizes HIV testing results from the second cycle of NHBS, conducted in 2010, which focused on heterosexual persons with low socioeconomic status (SES) living in areas with high AIDS case rates. The results indicated that HIV prevalence was 2.3% overall and 1.1% among participants who did not report a previous positive HIV test result. Overall, 25.8% of participants had never been tested for HIV until the NHBS survey. Given the high HIV prevalence in this sample, additional research should be conducted to identify culturally appropriate interventions that overcome barriers to HIV testing and increase linkage to care for heterosexuals with low SES in urban areas with high prevalence of AIDS.

NHBS monitors HIV prevalence and HIV-associated behaviors among populations at high risk for acquiring HIV in 21 metropolitan statistical areas (MSAs) with high prevalence of AIDS. During 2010, NHBS collected data and conducted HIV testing among heterosexuals using respondent-driven sampling, a peer-referral sampling method. Because results from the pilot study in 2006–2007 demonstrated that persons with low SES* were more likely than persons with high SES to be infected, the 2010 cycle of NHBS focused on low SES populations (2,3).

Initial respondents selected from poverty areas† completed the survey and were asked to recruit up to five persons from their social networks. Their peers then completed the survey, and those who reported low SES and no injection drug use (IDU) in the preceding 12 months also were asked to recruit persons from their social networks. Men and women aged 18–60 years who resided in the MSA, had at least one sex partner of the opposite sex in the past 12 months, and were able to complete the survey in English or Spanish were eligible to participate. Using a standardized, anonymous questionnaire, participants were interviewed about sexual behaviors, drug use, HIV testing behaviors, and use of HIV prevention services.

All respondents were offered anonymous HIV testing, regardless of self-reported HIV infection status. HIV testing was performed by collecting blood or oral specimens for either conventional laboratory testing or point-of-contact rapid testing. A nonreactive rapid test was considered a negative test result. For persons with reactive rapid test results, final positive test results were determined based on supplemental Western blot or immunofluorescence assay. Participants received compensation for completing the survey and taking an HIV test and received incentives for recruiting their peers. Participants were included in this analysis if they reported low SES, completed the survey, consented to an HIV test, had a final positive or negative test result, and reported never engaging in male-male sex (for men) or IDU. The percentage of respondents who were HIV infected and did not report a previous positive HIV test result§ also was calculated, as a measure of undiagnosed HIV infection. Unweighted HIV prevalence estimates were calculated; although respondent-driven sampling can produce weighted estimates, the number of HIV infections in this analysis was too small to properly weight the estimates (4).

In 2010, a total of 12,478 persons were screened for participation in NHBS, of whom 11,114 (89.1%) were eligible. Of these, 8,473 (76.2%) met criteria for inclusion in this analysis.¶ Median age for participants was 33 years; 61.9% were aged 18–39 years. The majority (71.9%) of participants were black, 36.2% had less than a high school education, and 62.5% reported an annual household income of less than $10,000.

Among the 8,473 participants, 197 (2.3%) tested positive for HIV infection, and prevalence was similar for men (2.2%) and women (2.5%) (Table 1). HIV prevalence was 2.8% among blacks and 1.2% among Hispanics or Latinos. Prevalence was higher for participants who reported less than a high school education (3.1%), compared with those with a high school education (1.8%). Prevalence also was higher for those with an annual household income less than $10,000 (2.8%), compared with those with an income of $20,000 or more (1.2%) and for those reporting having an exchange sex partner** in the past 12 months (3.7%) versus those not reporting an exchange sex partner (2.1%). Prevalence also was higher for those reporting using crack cocaine in the past 12 months (6.3%) compared with those not reporting crack cocaine use (1.8%). Prevalence was highest among those living in participating MSAs in the Northeast (4.1%) and South (3.9%) regions of the United States.

What is already known on this topic?

An estimated 27% of prevalent human immunodeficiency virus (HIV) infections in the United States are attributed to heterosexual contact. Heterosexuals with a low socioeconomic status (SES) are disproportionately more likely to be infected with HIV.

What is added by this report?

Low-SES heterosexuals in metropolitan statistical areas (MSAs) with a high acquired immunodeficiency syndrome (AIDS) prevalence were recruited by the National HIV Behavioral Surveillance System (NHBS) for interviews and HIV testing. Of 8,473 persons tested, 197 (2.3%) were infected with HIV, with the highest prevalence of infection occurring among blacks, persons reporting crack cocaine use or exchange sex, those with low levels of education or income, and persons living in participating MSAs in the Northeast or South. Overall, 25.8% of participants had never been tested previously for HIV. Among participants who tested positive during the survey but did not report a previous positive HIV test, 36 (43.9%) said they had never had an HIV test before NHBS.

What are the implications for public health practice?

Efforts to prevent HIV among heterosexuals that include encouraging HIV testing among persons living in low SES communities in urban areas with high prevalence of AIDS are likely to have the greatest potential impact. It is particularly important to increase HIV testing and linkage to care among the heterosexual populations with the highest prevalence of HIV: blacks, persons who use crack cocaine or engage in exchange sex, and persons with low levels of income and education. Participating MSAs, particularly in the Northeast and South, are most likely to benefit from focused interventions among low-SES heterosexuals.

A total of 108 of the 8,473 participants reported a previous positive HIV test result. Among the 8,365 participants who did not report a previous positive HIV test result, 89 (1.1%) were HIV infected (Table 2). Among blacks, 1.3% were HIV infected, and among Hispanics or Latinos, 0.7% were HIV infected. The percentage of HIV infected was higher for participants who reported being unemployed (1.1%) or disabled (and unemployed) (2.7%), compared with employed (0.4%). Although the proportion who were HIV infected was similar among persons who had visited a health-care provider in the past year (1.1%) and those who had not (0.9%), it was higher among those who reported never being tested for HIV (1.6%) compared with being tested within the past 12 months (0.5%). The percentage who were HIV infected was higher for those who reported having an exchange sex partner in the past 12 months (2.0%) compared with not (0.9%) and using crack cocaine use in the past 12 months (2.6%) compared with not (0.9%) (Table 2). Among the 8,365, a total of 2,187 (26.1%) had never been tested for HIV; 3,417 (40.8%) reported that their last HIV test was >12 months ago, and 2,736 (32.7%) had been tested for HIV in the past 12 months (Table 2).

Among 82 participants†† who tested positive during NHBS, knew the date of their most recent HIV test, but did not report a previous positive HIV test result, 36 (43.9%) reported never having had an HIV test until NHBS. An additional 14 (17.1%) had been tested >5 years before the interview (Figure).

## Editorial Note

The findings from this analysis indicate that HIV prevalence among a sample of low-SES heterosexuals residing in MSAs with high AIDS prevalence was 2.3% overall and 1.1% among those who did not report a previous positive HIV test result. The overall 2.3% HIV prevalence among survey participants is approximately five times the 0.45% estimated for all persons aged ≥13 years in the United States (1). HIV prevalence was high among participants reporting exchange sex and crack cocaine use, those with less than a high school education, and those unemployed or disabled. These findings suggest the need for both behavioral and structural (5) HIV prevention interventions for these populations. Additional efforts should address reducing health inequities, particularly among African Americans and Hispanics or Latinos, two populations that comprised 91.7% of the NHBS participants.

Among the 1.1% who were infected with HIV but did not report a previous positive HIV test, 43.9% reported that they had never been tested for HIV infection until participating in NHBS. A key step to reducing the number of new HIV infections in the United States, as indicated in the National HIV/AIDS Strategy (6), is to increase the percentage of persons living with HIV who know their serostatus through HIV testing. Persons aware of their HIV infection often take steps to reduce their risk behaviors substantially and can be referred for treatment and care, which can reduce HIV transmission (7). Overall, among participants in this study, 25.8% had never been tested for HIV, underscoring the need for increased HIV testing and linkage to care for low-SES heterosexuals living in urban areas with a high prevalence of AIDS. CDC currently supports an expanded testing program to increase HIV testing among populations disproportionately affected by HIV in 30 health jurisdictions, including the 21 NHBS MSAs. In the first 3 years of this program, 2.8 million tests were conducted, and approximately 18,000 persons were newly diagnosed with HIV infection (8).

The findings in this report are subject to at least three limitations. First, some participants might not have accurately reported their HIV risk behaviors or previous HIV test results to interviewers, and results might be affected by social desirability bias. Second, sampling was limited to men and women who live in urban areas with a high prevalence of AIDS, and analyses were limited to those with low SES; findings might not be generalizable to other heterosexual groups. Finally, because of high levels of HIV stigma, poverty, and homelessness in this population, standard sampling methods were not considered practical; the data were not weighted to account for the complexities or potential biases of network-based sampling, and statistical tests were not conducted. Therefore, differences between groups should be interpreted with caution.

CDC and its partners are pursuing a high-impact prevention approach§§ to advance the goals of the National HIV/AIDS Strategy and maximize the effectiveness of current HIV prevention methods. This approach focuses on implementing prevention strategies that have shown the greatest potential to reduce new infections on a scale large enough to yield the greatest impact in populations and geographic areas with the greatest burden of disease. The high level of HIV infection observed in NHBS among low-SES heterosexuals living in MSAs with high AIDS prevalence is a serious public health concern. Efforts to 1) reduce stigma and make HIV testing accessible, affordable, and culturally acceptable (9); 2) improve linkage to HIV care and treatment; and 3) implement interventions that address behavioral and structural factors that place low-SES heterosexuals at higher risk for contracting HIV infection (6,9) could lead to reductions in HIV incidence and health inequities to achieve the goals of the National HIV/AIDS Strategy.

## Figures and Tables

**FIGURE:**
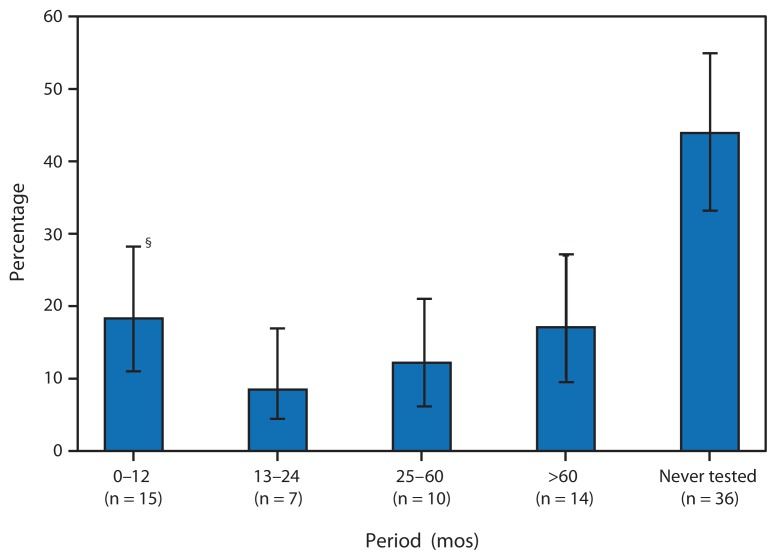
Period since most recent HIV test among HIV-infected heterosexuals at increased risk^*^ who did not report a previous positive HIV test result (n = 82^†^) — National HIV Behavioral Surveillance System, United States, 2010 **Abbreviation:** HIV = human immunodeficiency virus. ^*^Increased risk for HIV was defined as having low socioeconomic status (a household income below U.S. Department of Health and Human Services poverty guidelines [adjusted for household size] or a high school education or less). The analysis excluded persons who ever injected drugs and men who ever had sex with men. ^†^Among those reporting the date since their most recent HIV test. Excluded were seven participants who reported that their most recent HIV test was >12 months before the interview but did not report the year of that test. ^§^95% confidence interval.

**TABLE 1:** Prevalence of HIV infection among heterosexuals at increased risk (N = 8,473),* by selected characteristics — National HIV Behavioral Surveillance System, United States, 2010

Characteristic	No. tested		HIV prevalence		
	
	No.	(%)†	No.	(%)	(95% CI)
**Sex**					
Female	4,722	(55.7)	116	(2.5)	(2.0–2.9)
Male	3,751	(44.3)	81	(2.2)	(1.7–2.7)
**Age group (yrs)**					
18–24	2,445	(28.9)	—§	—	—
25–29	1,161	(13.7)	16	(1.4)	(0.8–2.2)
30–39	1,635	(19.3)	33	(2.0)	(1.4–2.8)
40–49	2,029	(23.9)	95	(4.7)	(3.8–5.6)
50–60	1,203	(14.2)	49	(4.1)	(3.0–5.4)
**Race/Ethnicity**					
Black, non-Hispanic	6,090	(71.9)	170	(2.8)	(2.4–3.2)
Hispanic or Latino	1,782	(21.0)	22	(1.2)	(0.8–1.9)
White, non-Hispanic	187	(2.2)	—	—	—
Other	406	(4.8)	—	—	—
**Highest level of education completed**					
Less than high school education	3,065	(36.2)	94	(3.1)	(2.5–3.7)
High school education or equivalent	4,129	(48.7)	76	(1.8)	(1.5–2.3)
Some college or more	1,278	(15.1)	27	(2.1)	(1.4–3.1)
**Annual household income**					
$0–$9,999	5,296	(62.5)	148	(2.8)	(2.4–3.3)
$10,000–$19,999	2,032	(24.0)	37	(1.8)	(1.3–2.5)
≥$20,000	1,040	(12.3)	12	(1.2)	(0.6–2.0)
**Poverty status**					
Above poverty guidelines	942	(11.1)	16	(1.7)	(1.0–2.7)
At or below poverty guidelines	7,426	(87.6)	181	(2.4)	(2.1–2.8)
**Employment status**					
Employed full time or part time	2,424	(28.6)	19	(0.8)	(0.5–1.2)
Unemployed	3,718	(43.9)	85	(2.3)	(1.8–2.8)
Disabled	881	(10.4)	67	(7.6)	(6.0–9.6)
Student	633	(7.5)	—	—	—
Other¶	816	(9.6)	23	(2.8)	(1.7–4.1)
**Region** **					
Northeast	1,629	(19.2)	67	(4.1)	(3.2–5.1)
South	2,714	(32.0)	105	(3.9)	(3.2–4.7)
Midwest	1,453	(17.1)	7	(0.5)	(0.2–1.0)
West	2,234	(26.4)	9	(0.4)	(0.2–0.8)
Territories	443	(5.2)	9	(2.0)	(1.0–3.8)
**Health coverage**					
No coverage	3,856	(45.5)	55	(1.4)	(1.1–1.9)
Private health insurance or HMO	615	(7.3)	—	—	—
Government program	3,814	(45.0)	135	(3.5)	(3.0–4.1)
Other coverage (includes multiple coverage)	163	(1.9)	—	—	—
**Exchange sex partner in past 12 months** ††					
Yes	1,410	(16.6)	52	(3.7)	(2.8–4.8)
No	7,063	(83.4)	145	(2.1)	(1.7–2.4)
**Crack cocaine use in past 12 months**					
Yes	1,007	(11.9)	63	(6.3)	(4.8–7.9)
No	7,466	(88.1)	134	(1.8)	(1.5–2.1)
**Total**	8,473	(100.0)	197	(2.3)	(2.0–2.7)

**Abbreviations:** HIV= human immunodeficiency virus; CI = confidence interval; HMO = health maintenance organization.

* Increased risk for HIV was defined as having low socioeconomic status (a household income below U.S. Department of Health and Human Services poverty guidelines [adjusted for household size] or a high school education or less). The analysis excluded persons who ever injected drugs and men who ever had sex with men.

† Totals might not add to 100% because of missing data.

§ Data suppressed because the number or numerator was five or fewer.

¶ Includes homemaker and retired.

** The U.S. Census regions in which the 21 metropolitan statistical areas of the National HIV Behavioral Surveillance System are located. The Northeast region consists of Boston, Massachusetts; Nassau-Suffolk Counties, New York; New York, New York; Newark, New Jersey; and Philadelphia, Pennsylvania. The South region consists of Atlanta, Georgia; Baltimore, Maryland; Dallas, Texas; Houston, Texas; Miami, Florida; New Orleans, Louisiana; and Washington, District of Columbia. The Midwest region consists of Chicago, Illinois; Detroit, Michigan; and St. Louis, Missouri. The West region consists of Denver, Colorado; Los Angeles, California; San Diego, California; San Francisco, California; and Seattle, Washington. The Territories consists of San Juan, Puerto Rico.

†† An exchange sex partner was defined as someone the participant gave things such as money or drugs to in exchange for sex or someone who gave the participant things such as money or drugs in exchange for sex.

**TABLE 2:** Prevalence of HIV infection among heterosexuals at increased risk* who did not report a previous positive HIV test result (n = 8,365), by selected characteristics — National HIV Behavioral Surveillance System, United States, 2010

Characteristic	No. tested	HIV prevalence		

		No.	(%)	(95% CI)
**Sex**				
Female	4,655	49	(1.1)	(0.8–1.4)
Male	3,710	40	(1.1)	(0.8–1.5)
**Age group (yrs)**				
18–24	2,443	—†	—	—
25–29	1,155	10	(0.9)	(0.4–1.6)
30–39	1,618	16	(1.0)	(0.6–1.6)
40–49	1,972	38	(1.9)	(1.4–2.6)
50–60	1,177	23	(2.0)	(1.2–2.9)
**Race/Ethnicity**				
Black, non-Hispanic	5,995	75	(1.3)	(1.0–1.6)
Hispanic or Latino	1,772	12	(0.7)	(0.4–1.2)
White, non-Hispanic	187	—	—	—
Other	403	—	—	—
**Highest level of education**				
Less than high school education	3,012	41	(1.4)	(1.0–1.8)
High school education or equivalent	4,087	34	(0.8)	(0.6–1.2)
Some college or more	1,265	14	(1.1)	(0.6–1.8)
**Annual household income**				
$0–$9,999	5,213	65	(1.2)	(1.0–1.6)
$10,000–$19,999	2,015	20	(1.0)	(0.6–1.5)
≥$20,000	1,032	—	—	—
**Employment status**				
Employed full time or part time	2,414	9	(0.4)	(0.2–0.7)
Unemployed	3,672	39	(1.1)	(0.8–1.4)
Disabled	837	23	(2.7)	(1.8–4.1)
Student	630	—	—	—
Other§	811	18	(2.2)	(1.3–3.5)
**Region** ¶				
Northeast	1,591	29	(1.8)	(1.2–2.6)
South	2,649	40	(1.5)	(1.1–2.1)
Midwest	1,453	7	(0.5)	(0.2–1.0)
West	2,229	—	—	—
Territories	443	9	(2.0)	(1.0–3.8)
**Health coverage**				
No coverage	3,827	26	(0.7)	(0.4–1.0)
Private health insurance or HMO	615	—	(0.8)	(0.3–1.9)
Government program	3,736	57	(1.5)	(1.2–2.0)
Other coverage (includes multiple coverage)	162	—	—	—
**Visited health-care provider** **				
Yes	5,692	64	(1.1)	(0.9–1.4)
No	2,669	25	(0.9)	(0.6–1.4)
**Had an STD diagnosis** ††				
Yes	839	9	(1.1)	(0.5–2.0)
No	7,526	80	(1.1)	(0.8–1.3)
**Previous HIV test**				
Never tested	2,187	36	(1.6)	(1.2–2.3)
>12 months ago	3,417	37	(1.1)	(0.8–1.5)
≤12 months ago	2,736	15	(0.5)	(0.3–0.9)
**Exchange sex partner in past 12 months** §§				
Yes	1,386	28	(2.0)	(1.4–2.9)
No	6,979	61	(0.9)	(0.7–1.1)
**Crack cocaine use in past 12 months**				
Yes	969	25	(2.6)	(1.7–3.8)
No	7,396	64	(0.9)	(0.7–1.1)
**Total**	**8365**	**89**	**(1.1)**	**(0.9–1.3)**

**Abbreviations:** HIV= human immunodeficiency virus; CI = confidence interval; HMO = health maintenance organization; STD = sexually transmitted disease.

* Increased risk for HIV was defined as having low socioeconomic status (a household income below U.S. Department of Health and Human Services poverty guidelines [adjusted for household size] or a high school education or less). The analysis excluded persons who ever injected drugs and men who ever had sex with men.

† Data suppressed because the number or numerator was five or fewer.

§ Includes homemaker and retired.

¶ The U.S. Census regions in which the 21 metropolitan statistical areas of the National HIV Behavioral Surveillance System are located. The Northeast region consists of Boston, Massachusetts; Nassau-Suffolk Counties, New York; New York, New York; Newark, New Jersey; and Philadelphia, Pennsylvania. The South region consists of Atlanta, Georgia; Baltimore, Maryland; Dallas, Texas; Houston, Texas; Miami, Florida; New Orleans, Louisiana; and Washington, District of Columbia. The Midwest region consists of Chicago, Illinois; Detroit, Michigan; and St. Louis, Missouri. The West region consists of Denver, Colorado; Los Angeles, California; San Diego, California; San Francisco, California; and Seattle, Washington. The Territories consists of San Juan, Puerto Rico.

** Visited a doctor, nurse, or other health-care provider in the past 12 months.

†† Participant self-reported diagnosis by a health-care provider of any STD in 12 months preceding interview.

§§ An exchange sex partner was defined as someone the participant gave things like money or drugs to in exchange for sex or someone who gave the participant things like money or drugs in exchange for sex.

## Footnotes

* Low SES was defined as having a household income (adjusted for household size) at or below the poverty level guidelines or no more than a high school education. Additional information available at http://aspe.hhs.gov/poverty.

† Poverty areas are defined by the U.S. Census Bureau as census tracts in which 20% or more of the residents live below the poverty threshold. Additional information available at http://www.census.gov/hhes/www/poverty/methods/definitions.html.

§ Persons not reporting a previous positive HIV test result included those who reported that their most recent HIV test result was negative, indeterminate, or unknown, or that they had never been tested.

¶ Persons were excluded from the analysis for the following reasons (categories are not mutually exclusive): 715 did not report a low SES, 1,339 reported IDU in the past 12 months, 753 men reported male-male sex, and 262 persons had no record of consent for the NHBS HIV testing, indeterminate or discordant NHBS HIV test results (i.e., they reported being HIV-positive but had a negative or indeterminate test result), or invalid NHBS HIV test results.

** An exchange sex partner was defined as someone the participant gave things such as money or drugs to in exchange for sex or someone who gave the participant things such as money or drugs in exchange for sex.

†† Excludes seven participants who reported that their most recent HIV test was >12 months before the interview but did not report the year of that test.

§§ Additional information available at http://www.cdc.gov/nchhstp/newsroom/hivfactsheets/future/high-impact-prevention.htm.
